# Transcriptome analysis reveals the potential mechanism of the response to scale insects in *Camellia sasanqua* Thunb

**DOI:** 10.1186/s12864-024-09980-y

**Published:** 2024-01-24

**Authors:** Hongye Zhang, Xubo Wang, Ziyun Yang, Yan Bai, Longqing Chen, Tian Wu

**Affiliations:** 1 School of Landscape Architecture and Horticulture Sciences, Southwest Forestry University, Kunming, 650224 China; 2 Yunnan Biodiversity Research Institute, Southwest Forestry University, Kunming, 650224 China

**Keywords:** RNA-Seq, Differentially expressed genes, Plant hormone, MAPK signaling, Transcription factor, *Pseudaulacaspis sasakawai* Takagi, Ornamental plant

## Abstract

**Background:**

*Camellia sasanqua* Thunb. is an essential woody ornamental plant. Our continuous observation found that scale insects often infest *C. sasanqua* all year round in Kunming, China, resulting in poor growth. Scientifically preventing and controlling the infestation of scale insects should be paid attention to, and the mechanism of scale insects influencing *C. sasanqua* should be used as the research basis.

**Results:**

The scale insect was identified as *Pseudaulacaspis sasakawai* Takagi. We analyzed transcriptome sequencing data from leaves of *C. sasanqua* infested with scale insects. A total of 1320 genes were either up-regulated or down-regulated and differed significantly in response to scale insects. GO (Gene Ontology) annotation analysis showed that the pathway of catalytic activity, binding, membrane part, cell part, and cellular process were affected. KEGG (Kyoto Encyclopedia of Genes and Genomes) pathway analysis showed that most DEGs (differentially expressed genes) involved in plant hormone signal transduction, MAPK signaling pathway, flavonoid biosynthesis, tropane, piperidine and pyridine alkaloid biosynthesis. We also observed that the expression of galactose metabolism and carotenoid biosynthesis were significantly influenced. In addition, qRT-PCR (quantitative real-time PCR) validated the expression patterns of DEGs, which showed an excellent agreement with the transcriptome sequencing.

**Conclusions:**

Our transcriptomic analysis revealed that the *C. sasanqua* had an intricate resistance strategy to cope with scale insect attacks. After sensing the attack signal of scale insects, *C. sasanqua* activated the early signal MAPK (mitogen-activated protein kinase) to activate further transcription factors and Auxin, ET, JA, ABA, and other plant hormone signaling pathways, ultimately leading to the accumulation of lignin, scopolin, flavonoids and other secondary metabolites, produces direct and indirect resistance to scale insects. Our results suggested that it provided some potential resources of defense genes that would benefit the following resistance breeding in *C. sasanqua* to scale insects.

**Supplementary Information:**

The online version contains supplementary material available at 10.1186/s12864-024-09980-y.

## Introduction

*C. sasanqua* is a popular ornamental Camellia species globally. *C. sasanqua* is considerably attractive owing to its wide range of forms and winter aesthetic value [1]. Despite this value, the function provided is greatly compromised by injurious insects that frequently outbreak in urban landscapes [2, 3]. One of the most destructive pests on tea plantations, the scale insect, has yet to be well studied. The scale insect, a typical sucking sap insect, mainly sucks juice from the leaves, reducing photosynthesis and respiration and affecting the average growth of host plants [4, 5]. These scale insects are challenging to eradicate. On the one hand, the surfaces protective layer enhances scale insects resistance to the surroundings. On the other hand, due to their short generation times and high reproductive rates. Agrochemical applications are the most common means of controlling scale insects. However, they are often associated with several adverse side effects, including pesticide resistance, secondary pest outbreaks, and harmful effects on human health and the environment. Breeding insect and disease-resistant cultivars is the most environmentally benign and sustainable method of plant protection.

Studying how plants respond to scale insects can provide a new perspective to reveal the insect-resistant mechanism. Insects frequently attack plants. Therefore, they evolved elaborate defense systems during the last 450 million years of co-evolution [5]. when sap-sucking insect feed on plants, their mouthparts penetrated plant cell walls, and secreted gelling and watery saliva from their salivary glands. Plant can recognize this insects oral secretionon, frass, ovipositional fluids, and the endosymbionts [6]. Receptors that activated early signaling components such as Ca^2+^, reactive oxygen species (ROS), and mitogen-activated protein kinase (MAPK) [7]. Meanwhile, The early signaling was transmitted by plenty of a lot of pathways on the plant, such as JA, ET, ABA, SA. One or more plant defense hormones were accumulated, and induced a local or systematic defense response. Plants experience physiological function some changes when subjected to insect attacks, such as gene expression, the accumulation of secondary metabolites. On the other hand, insects secrete various types of effectors to interfere with plant defense at multiple levels for better adaptation [8]. It was a complicated and hard-fought competition.

Meanwhile, these changes asked plants to require careful consideration and trade-offs between growth and defense. This means that plants might need reducing damage caused by insects with the biological processes of optimizing vegetative growth [9, 10]. Seed reatment with JA induces resistance to water weevil but reduces plant growth and yield in rice, *Oryza sativa* [11]. Transcription factors also led to significant alteration of both defensive metabolites and insect performance. This was shown in *Arabidopsis* and rice [12]. However, plant resistance insects would need more information on molecular mechanisms. This study investigated changes in the transcriptome data induced by *C. sasanqua* by scale insects. Exploring the molecular mechanism of the *C. sasanqua* response and screening critical genes related to scale insect regulation could provide a theoretical reference for cultivating resistant germplasm.

## Results

### Identification of the scale insects based on growth rule and microexamination

**Fig. 1:**
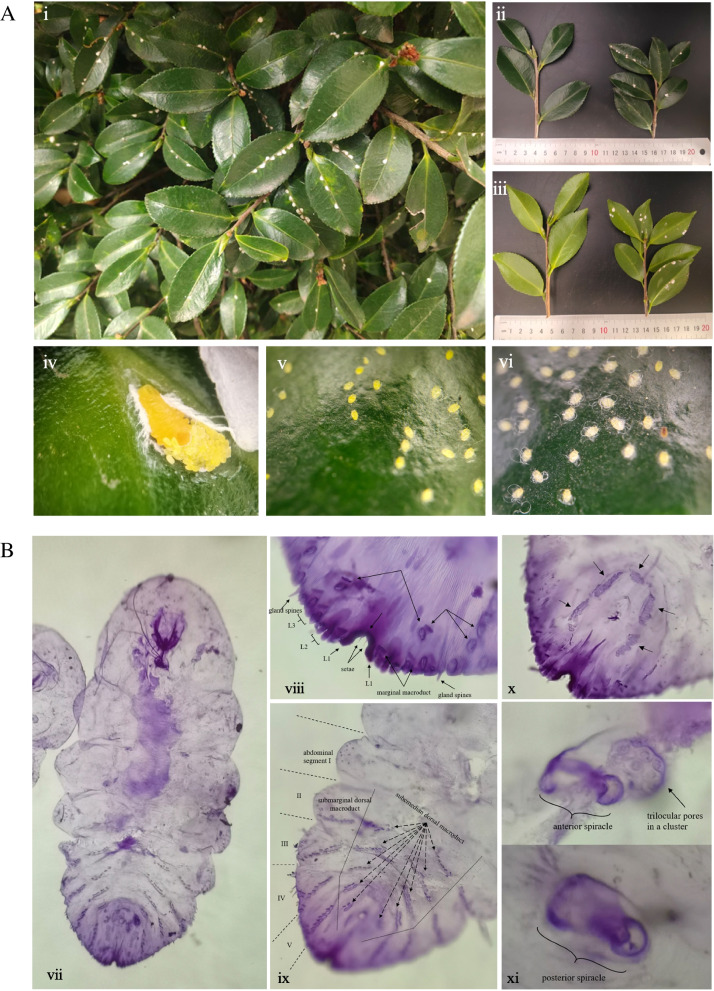
Observation on the status of *C. sasanqua* infected by scale insects. **A **Scale insects infested the *C. sasanqua.* i. the scale insects occurred in the wild; ii. the comparison of normal leaves and leaves infected by scale insects (front); iii. The comparison of normal leaves and leaves infected by scale insects (reverse side); iv. the status of scale insects in June; v. the status of scale insects in July; vi. the status of scale insects in August. **B **Identification of scale insects. vii xi slide-mounted specimens. vii. observing the adult female on the sheet glass; viii. the feature of pygidium; ix. the arrangement of dorsal macroduct; x. perivulvar pores in 5 groups; xi. detail of spiracle

The white covers were found on the leaves of *C*. *sasanqua* (Fig. 1A i), on the front and side of the leaves (Fig. 1A ii, iii). When peeled off the white cover, the female and yellow suborbicular eggs were exposed (Fig. 1A iv). The slide specimen of the scale insects was observed under a microscope (Fig. 1B vii). The pygidium usually with gland spines, median lobes (L1) without gland spines, second lobes (L2) bilobate, Third lobes (L3) well-developed, dorsal macroducts double bolt type, the dorsal macroducts about the same size as marginal macroducts (Fig. 1B viii). The dorsal macroducts on the submedian and submarginal area of abdominal segments II-VI (Fig. 1B ix), five groups of perineal glands (Fig. 1B x). The anterior spiracle with trilocular pores, and the posterior spiracle does without trilocular pores (Fig. 1B xi). Therefore, the scale insect was identified as *Pseudaulacaspis sasakawai* Takagi [13].

The generations of scale insects were different in each region. It observed that two ages can occur in a year in Kunming (Table 1). From early March to April each year, female adult insects lay eggs, and hatched eggs end in May. Hatching nymphs were stationary or utterly immobile on the plant, secreting filaments densely to coat the body, which ended in mid-June and entered the second instar nymphs stage, becoming adults (Fig. 1A v,vi). The second generation entered in July, and the new female adults began to lay eggs. The first instar nymphs appeared in August, and the second instar nymphs were found in September. In November, they overwinter as fertilized female adults until February of the following year.

**Table 1:** The life history of *Pseudaulacaspis sasakawai* Takagi

	Mar	Apr	May	Jun	Jul	Aug	Sep	Oct	Nov. Feb
First generation	♀	♀							
	○	○							
	1	1	1						
			2	2					
				♂	♂				
Second generation				♀	♀	♀			
					○	○			
					1	1			
						2	2		
							♂	♂	
							♀	♀	♀

**♀** females, **○** egg, 1 first instars, 2 s instars, **♂** males

### Validation of 17 DEGs

We selected 17 genes significantly expressed in the resistance pathway and tested them using quantitative real-time PCR (Fig S2). The results showed that the qRT-PCR expression patterns of genes and RNA-Seq were consistent, indicating the high reliability of the data.

### Transcriptome sequencing analyses

**Fig. 2:**
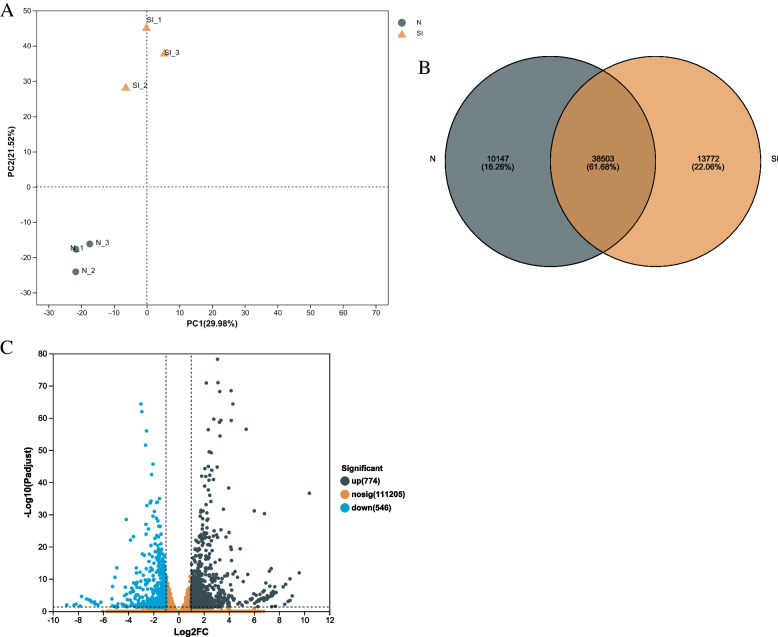
Analysis of transcriptome data of *C. sasanqua* infected by scale insects. **A **PCA analysis of normal leaves and leaves infested by scale insects. PC1, the first principal component; PC2, the second principal component. N, normal leaves; SI, leaves infested by scale insects. **B **A Venn diagram comparing the numbers of DEGs. **C **The genes of expression difference volcano map

Six samples were used for the transcriptomic analysis. After removing low-quality sequences, the high-throughput sequencing generated clean data for 41,533,976–46,731,398. The percentage of Q20 bases was 97.5% and above, the rate of Q30 bases was 93.4% and above, and GC content was between 44.83% and 45.39% (Table S1). After assembly, 15,353,475–17,773,208 clean reads were mapped to the transcript, accounting for 68.7–76.36%. The PCA (Principal component analysis) was performed on the samples of difference comparison (Fig. 2A). The distribution of each point shows that the separation trend between the groups was noticeable, there were differences between the sample groups, and the sample repeatability within the group was good. These data indicated that the sequencing results were accurate, covered most expressed genes, and could be analyzed in the subsequent steps.

### Analysis of differentially expressed genes

The total mapped reads of all genes were used for differential expression analysis using DESeq2. The *p*-value ≤ 0.05 was set as the threshold for DEGs. 10,147 genes were identified in normal leaves, and 13,772 genes were placed in infected leaves (Fig. 2B). Compared with normal leaves, A total of 1320 DEGs were identified in leaves of infects with scale insects, 774 (58%) up-regulated genes and 546 (41%) down-regulated genes (Fig. 2C), respectively. These differential genes were expressed in a range from -8 to tenfold. The number of up-regulated genes was significantly higher than that of down-regulated genes; we assumed that the up-regulated gene might play a more substantial role in the response of *C. sasanqua* to scale insects.

### GO function annotation analysis of DEGs

**Fig. 3:**
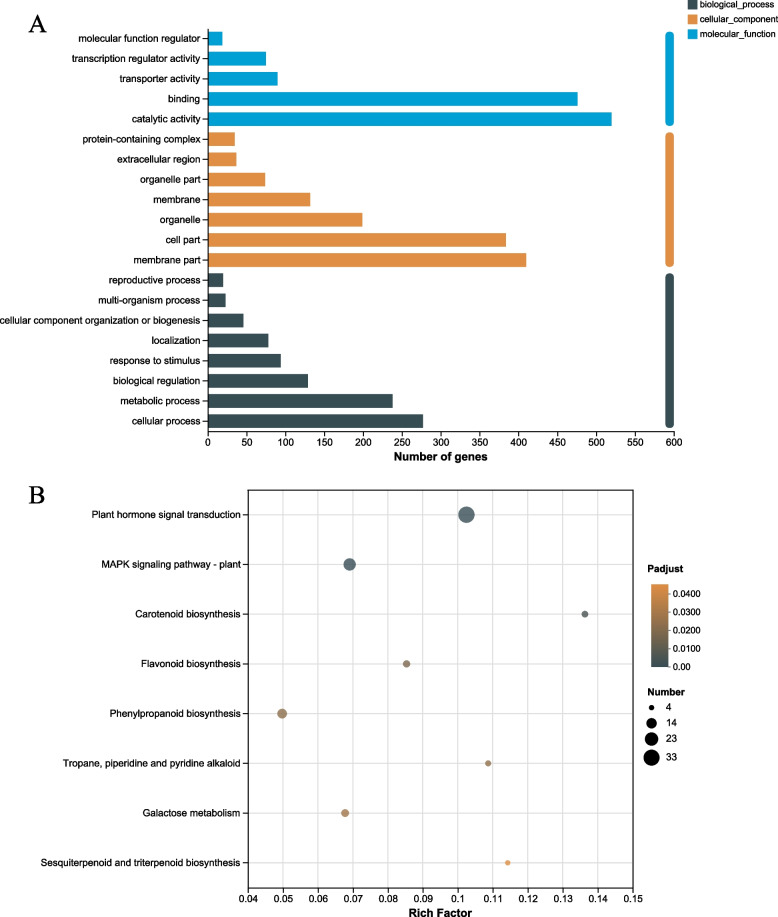
The analysis of GO annotation and KEGG enrichment. **A** The column diagram of GO annotation. **B** KEGG enrichment analysis scatter plot of the DEGs. The ordinate represents the KEGG pathway. The abscissa represents the Rich factor. The greater the Rich factor, the greater the degree of enrichment. The larger the dot, the higher the number of differential genes enriched by the pathway. Differences in pathways were considered significant when the *p* ≤ 0.05

One thousand three hundred twenty DEGs were successfully annotated to 20 functional groups, including eight biological processes, seven cellular components, and five molecular functions (Fig. 3A). In the molecular function category, the DEGs were mainly enriched in catalytic activity, binding, membrane part, cell part, and cellular process. These results indicated that when scale insects infested *C. sasanqua*, the plant defense system immediately responded to the stimulus, appropriately increased metabolic activities in the body, and produced defense substances to enhance the activities of various enzymes and promote defense.

### KEGG functional enrichment analysis of DEGs

The results showed that DEGs of the leaves infested with scale insects compared with normal leaves were categorized into eight pathways (Fig. 3B), which mainly included the plant hormone signal transduction (map04075), MAPK signaling pathway (map04016), carotenoid biosynthesis (map00906), flavonoid biosynthesis (map00941), galactose metabolism (map00052), tropane, piperidine, pyridine alkaloid biosynthesis (map00960), phenylpropanoid biosynthesis (map00940), and sesquiterpenoid and triterpenoid biosynthesis (map00909). The pathway analysis showed that differential expressed genes in the metabolic pathways induced by scale insects might be related to the induction of defense-related, such as plant hormone signal transduction and MAPK signaling pathway. These were the early signaling pathways of plants under biological stress. Flavonoid biosynthesis, phenylpropanoid biosynthesis, and tropane, piperidine, pyridine alkaloid biosynthesis are secondary metabolites that can respond to biological stress in time. Moreover, it should be focused on and deeply analyzed.

#### Analysis of DEGs related to MAPK signaling transduction

**Fig. 4:**
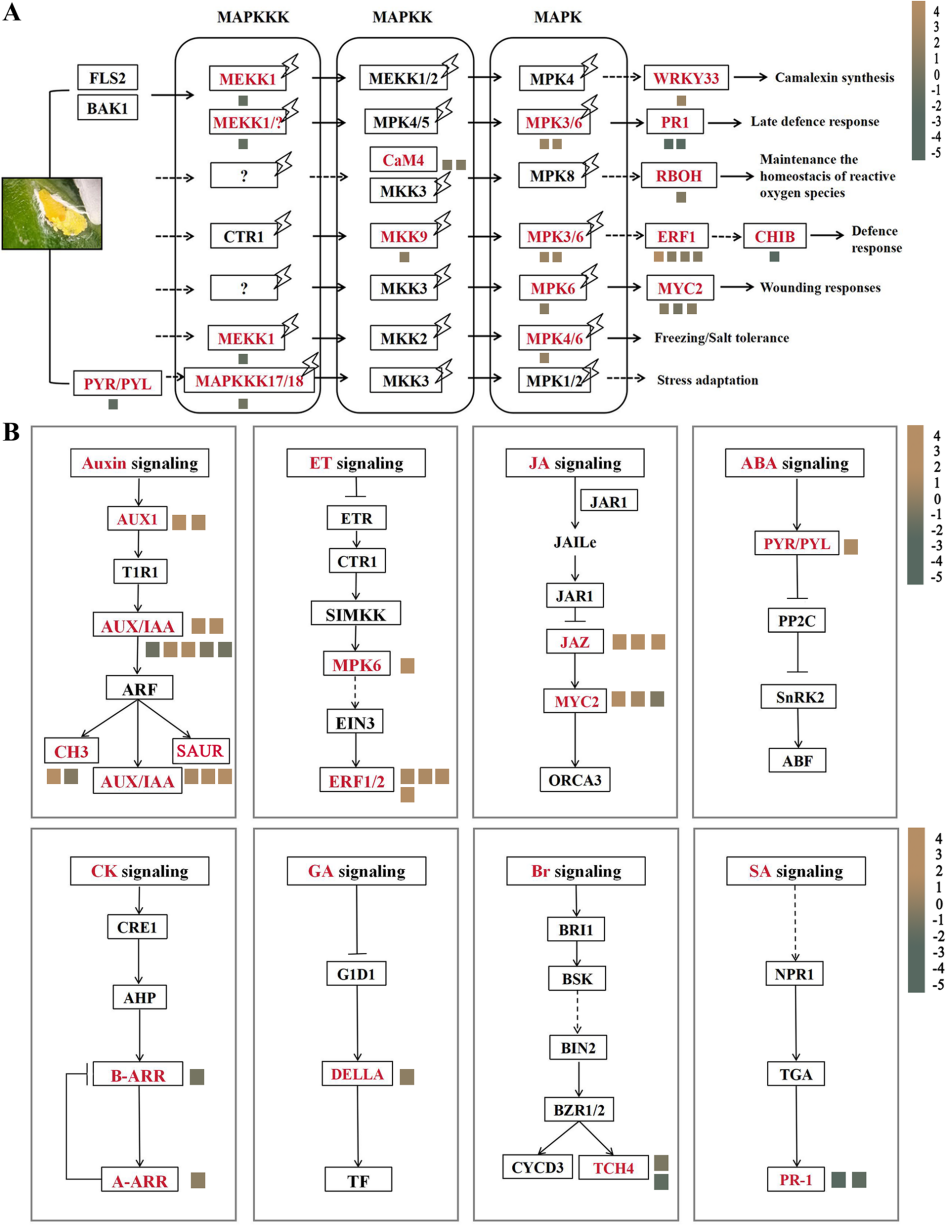
The genes related to MAPK and phytohormone signaling. **A** MAPK signaling pathway **B** Phytohormone signaling. Brown represents the up-regulated gene, and green represents the down-regulated gene. Color from brown to green represents Log2|FC| (≤ − 0.5 and ≥ 0.5) in descending order.  : phosphorylation

In comparison between normal and infected leaf groups, 19 DEGs were enriched in the MAPK signaling pathway (Fig. 4A). The MAPK genes were induced among them. The expression of *MEKK1* (mitogen-activated protein kinase kinase kinase 1), *PR-1* (pathogenesis-related protein 1) and, *CHIB* (chitinase) were considerably down-regulated. The expression of *MAPKKK17/18* (mitogen-activated protein kinase kinase kinase 17/18), *MPK3/6* (mitogen-activated protein kinase 3/6), *MPK6* (mitogen-activated protein kinase 6), *CaM4* (calmodulin-like protein), *MPK4/6* (mitogen-activated protein kinase 4/6), *ERF1* (ethylene-responsive transcription factor 1) were up-regulated, and *MKK9* (mitogen-activated protein kinase kinase 9) were slightly up-regulated. The *WRKY33* of the WRKY transcription factors family and *MYC2* of the MYC transcription factors were induced.

#### Analysis of DEGs related to plant hormones signaling

Plant hormones played a vital role in *C. sasanqua* against scale insects, and we found that 33 genes regulated eight hormone pathways. The 14 genes, including *AUX1*, *CH3* (auxin-responsive GH3 gene family), *SAUR* (SAUR family protein), and *AUX/IAA* (auxin-responsive protein IAA), were involved in the auxin signaling. In the ET signaling pathway (Fig. 4B), five significant up-regulation up-regulated genes involved *MPK6* and ERF. Six genes were identified in the JA signaling, including *JAZ* and transcription factor *MYC2*. *JAZ* and *MYC2* were up-regulated. Furthermore, scale insects could activate the other five plant hormone signaling pathways after the infection. Among them, the two genes involved *TCH4* (xyloglucan endotransglucosylase/hydrolase protein), *B-ARR* (two-component response regulator ARR-A family) were down-regulation, *PYR/PYL* (abscisic acid receptor PYR/PYL family) were up-regulated, *PR1* of the SA signaling was Significant down-regulated. These phytohormone signaling might cause cell wall modification and signal transduction through protein cascade, a rise in the defense gene expression levels, and an accumulation of defensive compounds.

#### Analysis of DEGs related to secondary metabolism

**Fig. 5:**
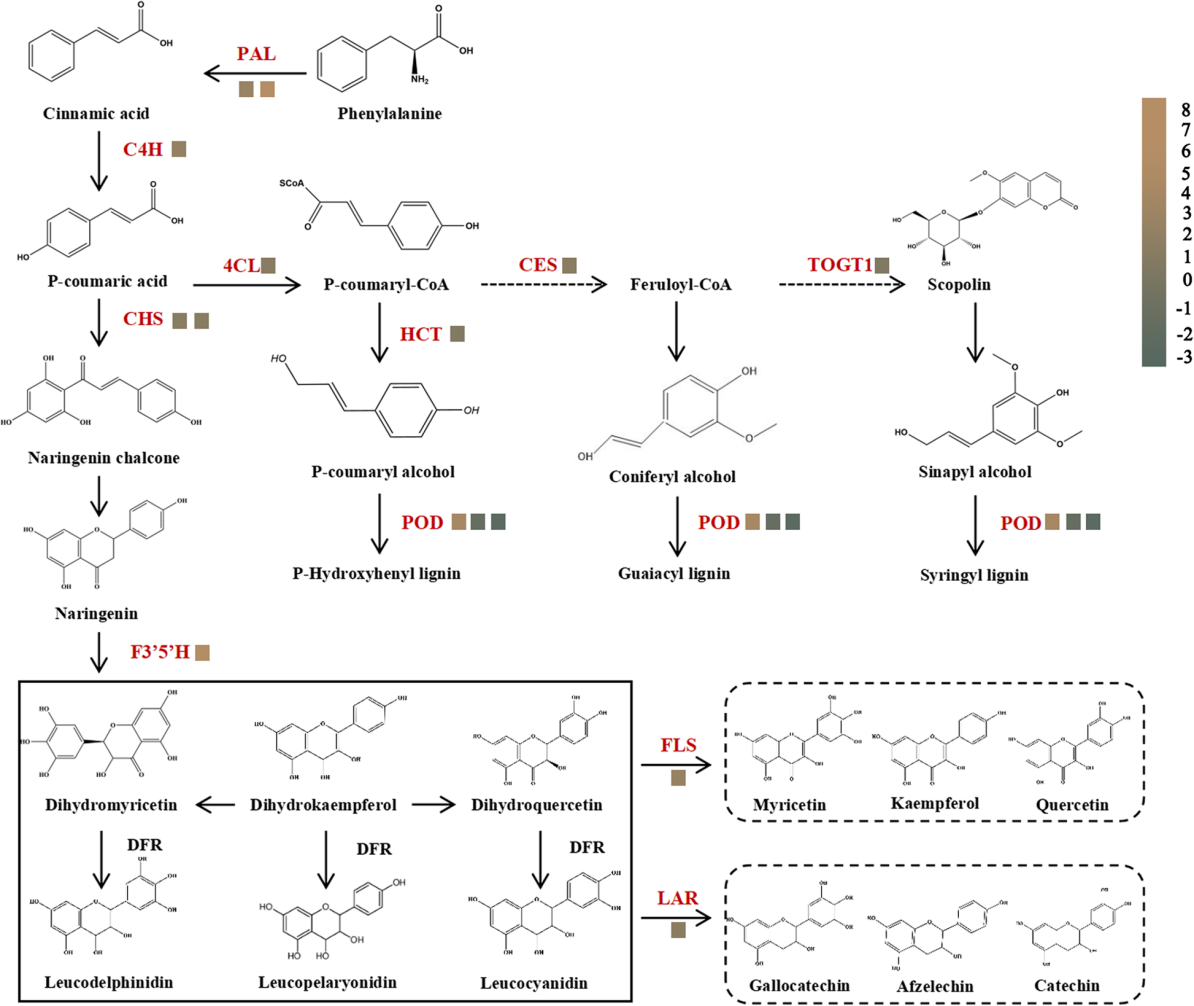
Expression of the genes involved in phenylalanine biosynthesis and flavonoid metabolism. PAL, phenylalanine ammonia-lyase; C4H, trans-cinnamic acid 4-monoxygenase; 4CL, 4-coumarate-CoA ligase; CHS, chalcone synthase; LAR, leucoanthocyanidin reductase; FLS, flavonol synthase; F3′5'H, flavonoid 3',5'-hydroxylase; COGT1, scopoletin glucosyl transferase; POD, peroxidase; HCT, hydroxy cinnamoyl transferase; All the genes showing Log2|FC| (≤ − 0.5 and ≥ 0.5) in expression were analyzed. Brown and green squares represent up- and down-regulated genes respectively

Our results showed that the genes related to phenylpropanoid biosynthesis and flavonoid metabolism pathways were induced. The seven genes associated with the flavonoid biosynthesis pathway were identified (Fig. 5), the expression of *CHS* (chalcone synthase), *LAR* (leucoanthocyanidin reductase), *FLS* (flavonol synthase), *C4H* (cinnamic acid 4-hydroxylase), F3′5’H (flavonoid 3′5’-hydroxylase) were significantly up-regulated. They produce afzelechin, catechin, gallocatechin, kaempferol, quercetin, and myricetin under the *LAR* and *FLS* converted. Furthermore, these are further modified to various flavonol derivatives. There were ten DEGs associated with the phenylpropanoid biosynthesis pathway identified that were all up-regulated (Fig. 5), including *PAL* (phenylalanine ammonia-lyase), *POD* (peroxidase), *C4H* (cinnamic acid 4-hydroxylase), *4CL* (4-coumarate-CoA ligase), *HCT* (hydroxy cinnamoyl transferase), *CSE* (caffeoylshikimate esterase), and *COGT1* (scopoletin glucosyltransferase). These genes led to the accumulation of syringyl lignin (S-lignin), guaiacol lignin (G-lignin), p-Hydroxypheny lignin (H-lignin), and scopolin. These metabolites would participate in the *C. sasanqua* response network.

#### Analysis of DEGs related to energy metabolism

**Fig. 6:**
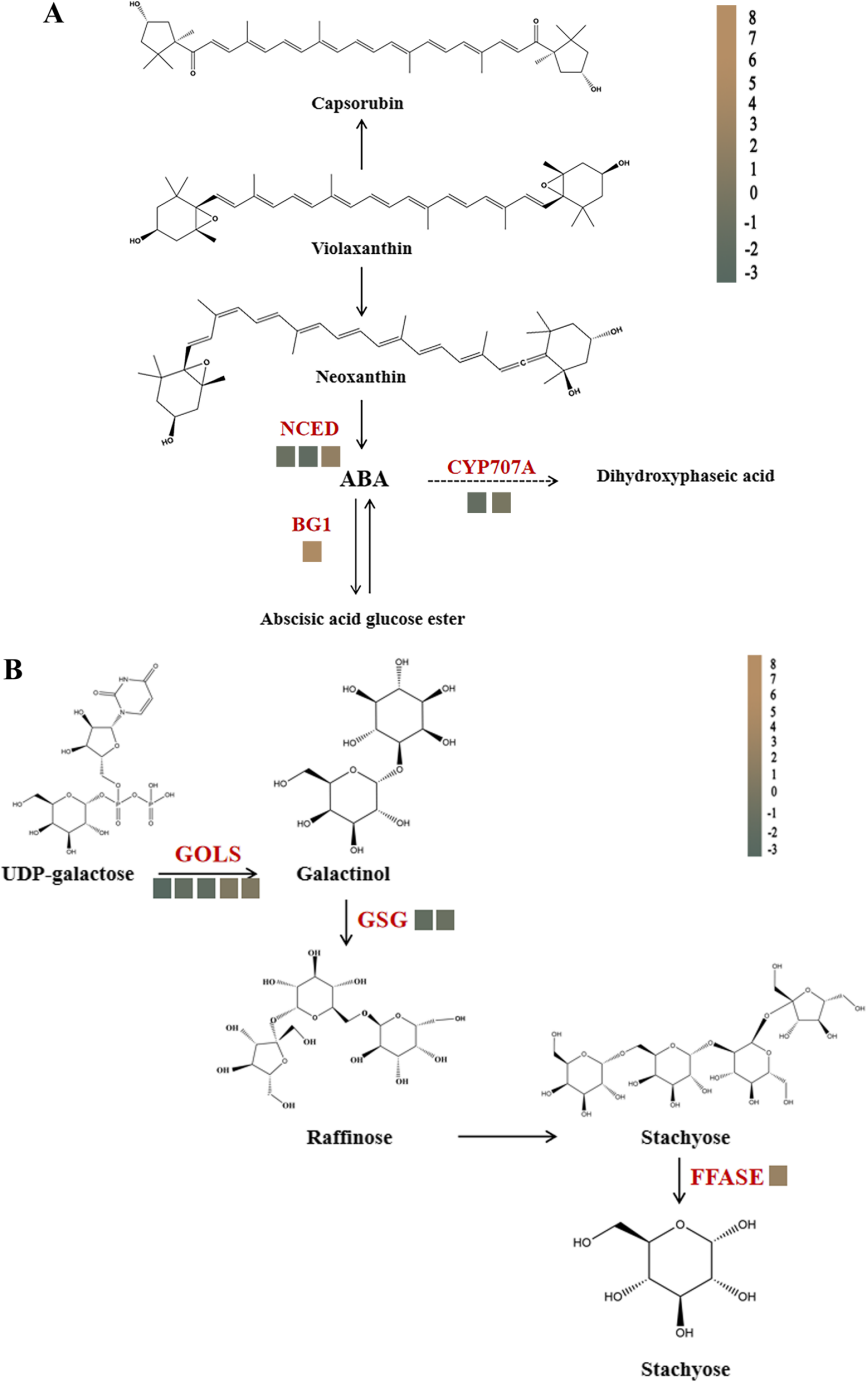
The Carotenoid and Galactose metabolism pathway related genes. **A** Carotenoid biosynthesis. NCED, 9-cis-epoxycarotenoid dioxygenase, CYP707A, abscisic acid 8'-hydroxylase, BG1, beta-glucosidase. **B** Galactose metabolism. GOLS, galactinol synthase, GSG, galactinol-sucrose galactosyltransferase, FFASE, beta-fructofuranosidase

The results showed that six genes related to carotenoid biosynthesis could be induced (Fig. 6A), *NCED* (9-cis-epoxycarotenoid dioxygenase), *CYP707A* (abscisic acid 8'-hydroxylase), and *BG1* (beta-glucosidase). Three *NCED* were identified, with two genes down-regulated and one gene up-regulated, *CYP707A* were down-regulated, and *BG1* showed significant up-regulation. Eight genes were identified in galactose metabolism (Fig. 6B), including galactinol synthase (*GOLS*) and *GSG* (galactinol-sucrose galactosyltransferase), *FFASE* (beta-fructofuranosidase). Two genes were slightly up-regulated, three significant were down-regulated in *GOLS*, and *GSG* showed significant down-regulation.

### The transcription regulatory factors were induced

**Fig. 7:**
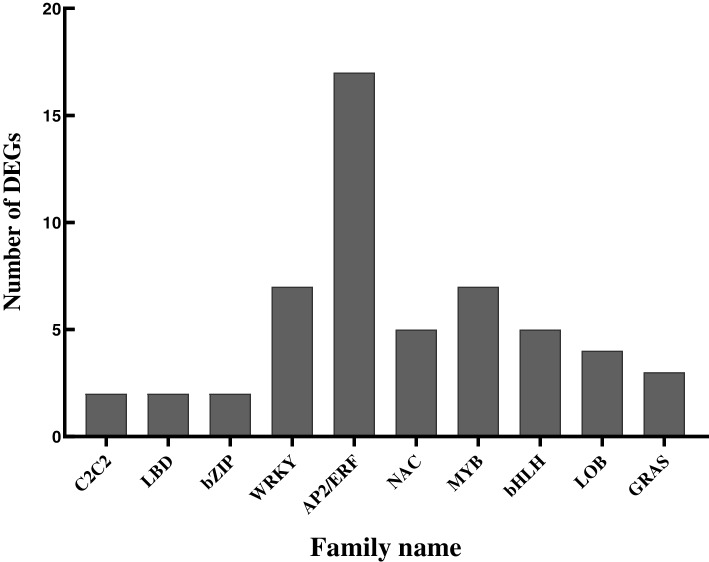
The transcription factor Family were induced

These ten transcription factors (TFs) families were regulated after being stimulated by a scale insects (Fig. 7). Among these families, 17 genes belonging to the AP2/ERF transcription factor family were up-regulated. Seven genes of the WRKY transcription factor family and seven of the MYB transcription factor family were identified, six up-regulated and one down-regulated. Five genes of the NAC family had been found, two up-regulated and three down-regulated (Table 2). The transcription factors of the WRKY, MYB, AP2/ERF, and NAC families were reported as essential components in plant disease resistance.

**Table 2:** Transcription factors potentially involved in scale insect resistance regulation

Gene family	Gene_id	Description	E-value	Log2FC	P-adjust
AP2/ERF	TRINITY_DN8836_c0_g1	ERF 5	7.50E-16	2.45	5.51
	TRINITY_DN3571_c0_g1	ERF109	1.90E-15	1.63	4.72
	TRINITY_DN15462_c0_g1	ERF 17	2.70E-12	4.19	6.00
	TRINITY_DN42648_c0_g1	ERF 27	2.00E-11	1.07	0.04
	TRINITY_DN29195_c0_g1	ERF1B	2.60E-13	1.73	0.00
	TRINITY_DN43295_c0_g1	ERF1B	9.40E-15	2.36	6.23
	TRINITY_DN4389_c3_g1	APETALA2	7.10E-15	1.40	2.09
	TRINITY_DN4389_c2_g1	ERF PAP2-7	5.60E-15	2.56	8.98
	TRINITY_DN10918_c0_g1	DREB2C	2.40E-13	1.38	0.01
	TRINITY_DN8309_c0_g1	ERF 4	1.60E-13	1.02	6.84
	TRINITY_DN195_c0_g2	APETALA2	2.20E-18	2.33	4.77
	TRINITY_DN26224_c0_g1	ERF1B	8.20E-15	5.33	0.00
	TRINITY_DN12680_c0_g1	PTI5	1.00E-14	2.77	4.06
	TRINITY_DN6451_c1_g1	ERF1B	3.80E-13	2.64	0.00
	TRINITY_DN7158_c0_g1	ERF	1.10E-14	2.75	4.30
	TRINITY_DN10522_c0_g1	ERF2	9.40E-16	1.54	1.22
	TRINITY_DN10522_c0_g2	ERF2	4.80E-13	6.16	9.90
MYB	TRINITY_DN16759_c0_g1	MYB44	2.60E-34	-1.03	1.08
	TRINITY_DN35609_c0_g1	MYB4	6.70E-31	1.88	2.11
	TRINITY_DN11221_c0_g1	MYB308	1.40E-28	3.44	2.61
	TRINITY_DN9246_c0_g1	MYB24	6.30E-25	2.47	1.07
	TRINITY_DN21561_c0_g1	MYB4	3.50E-32	2.23	2.21
	TRINITY_DN34023_c0_g1	MYB77	7.80E-33	1.21	0.01
	TRINITY_DN8048_c0_g1	MYB4	1.80E-06	1.35	1.56
WRKY	TRINITY_DN12474_c0_g1	WRKY75	3.00E-26	4.02	0.02
	TRINITY_DN6194_c0_g1	WRKY43	1.90E-24	3.33	0.01
	TRINITY_DN28643_c0_g1	WRKY41	4.10E-27	1.37	1.88
	TRINITY_DN14122_c0_g1	WRKY33	3.80E-32	3.06	1.83
	TRINITY_DN5614_c1_g3	WRKY75	5.20E-26	1.30	0.00
	TRINITY_DN12229_c0_g1	WRKY24	1.20E-23	3.49	7.16
	TRINITY_DN956_c0_g3	WRKY40	1.80E-24	-2.61	6.08
bHLH	TRINITY_DN12489_c0_g1	bHLH107	8.90E-12	1.60	0.00
	TRINITY_DN11800_c0_g1	bHLH162	4.70E-07	-2.08	0.00
	TRINITY_DN16884_c0_g1	bHLH041	1.70E-12	3.00	2.83
	TRINITY_DN4449_c0_g1	BIM2	3.40E-16	1.77	9.54
	TRINITY_DN14924_c0_g1	bHLH92	2.40E-06	2.45	4.54
NAC	TRINITY_DN17029_c0_g2	NAC2	3.70E-39	2.41	2.17
	TRINITY_DN15078_c0_g1	NAC83	2.00E-31	1.37	2.12
	TRINITY_DN2542_c0_g1	NAC7	3.30E-10	-1.03	1.39
	TRINITY_DN7592_c0_g1	NAC22	1.00E-34	-1.09	1.05
	TRINITY_DN4749_c0_g1	NAC26	4.50E-31	-1.15	1.00
bZIP	TRINITY_DN21948_c0_g2	bZIP53	4.60E-12	2.15	5.79
	TRINITY_DN14751_c0_g1	bZIP60	1.30E-07	-1.54	1.13
LOB	TRINITY_DN9176_c0_g1	LOB1	3.10E-41	1.91	0.00
	TRINITY_DN950_c0_g1	LOB41	6.50E-24	-2.06	7.86
	TRINITY_DN950_c0_g2	LOB41	6.00E-24	-3.30	9.56
	TRINITY_DN950_c0_g3	LOB41	2.80E-06	-2.21	0.00
GRAS	TRINITY_DN12540_c0_g1	SCL33	2.30E-113	2.18	9.92
	TRINITY_DN49748_c2_g1	RGL1	2.80E-129	-3.31	3.60
	TRINITY_DN3084_c3_g1	SLR1	7.60E-126	1.00	0.00
C2C2	TRINITY_DN5829_c0_g1	GATA8	6.10E-15	-1.54	1.57
	TRINITY_DN5987_c0_g1	CDF3	7.30E-33	1.20	0.00
LBD	TRINITY_DN2747_c0_g1	ATHB-12	8.90E-17	2.36	1.21
	TRINITY_DN26223_c0_g1	HOX11	1.00E-15	1.31	0.01

### Anthocyanins content in leaves after scale insects attack

**Fig. 8:**
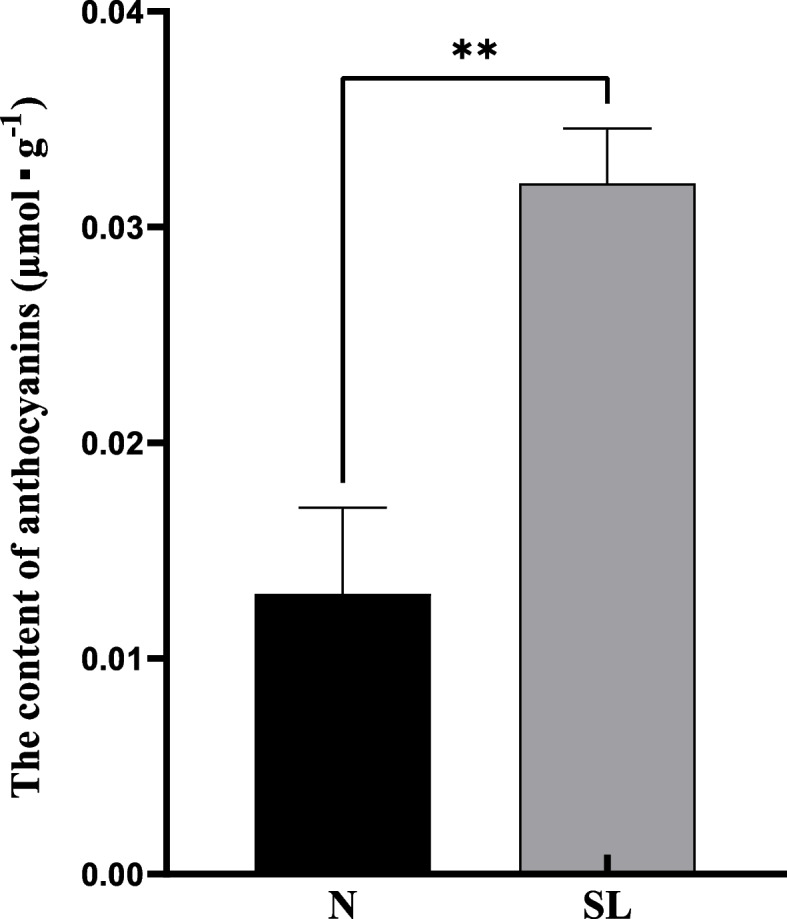
Determination of anthocyanin content. N: normal leaves, SL: the leaves infected by scale insects

After measuring the anthocyanin content of normal leaves (N) and the leaves infected by scale insects (SL), we found that the anthocyanin content of leaves infected by scale insects was 2.4 folds higher than that of normal leaves (Fig. 8), indicating that the anthocyanin content of the leaves attacked by scale insects was increased.

## Discussion

In this research, we found marked differences between samples by KEGG, the related gene of plant hormones, MAPK signals transductions, carotenoid biosynthesis, flavonoid biosynthesis, phenylpropanoid biosynthesis, and galactose metabolism, showing significant changes. The defense system composed of plant defense response might be the elaborate signaling networks and the sophisticated crosstalk occurring among the different pathways.

### MAPK signals and plant hormones played a role in the insistence of scale insects

MAPKs are essential in plant defense against insects, as they control the accumulation of plant hormones and the activity of TFs, and MAPKs often control the critical enzymes in secondary metabolite biosynthesis pathways [14]. For example, *OsMPK3/4* positively regulated striped stem borer (SSB) resistance in rice by mediating SA and JA signaling [15]. Several MAPK genes activated MAPK signaling pathways in our transcriptome data and showed significant changes, such as *MEKK1*, *MPK3/6*, and *MKK9*. In addition, we also found that *CaM4* and *CHIB* were involved in plant defense systems. We concluded that infestation of scale insects could activate the MAPK signaling pathway phosphorylate WRKY and induce plant defense response.

Plant hormones often play a central role in plant defense responses at almost all levels and transmit information by signaling cascades, enabling plants to survive challenging situations. In our study, Auxin, ET, ABA, and JA response genes were abundant and expressed at high levels in plant hormone metabolic pathways. Auxin was a crucial phytohormone that affected plant growth and development regulators [16]. The 14 genes expression of the Auxin signaling pathway was activated (Fig. 4B), including primary auxin-responsive genes of *AUX/IAA*, *GH3,* and *SAUR* gene families. Those were prominent auxin-responsive gene families. *Auxin /IAA* is an essential gene in the auxin family, which can regulate auxin signal transduction. The gene *SAUR* was involved in the maize insect resistance [17]. We speculated that *Aux/IAA*, *GH3,* and *SAUR* were involved in the resistance to scale insects by activating the Auxin signaling pathway in *C. sasanqua*.

Increasing the ET level or enhancing the ET signal might increase the resistance against insects in various plant species. In our results, one *MPK6* and four *ERF1/2* were significantly up-regulated (Fig. 4B). ERFs were important plant-specific transcription factors regulating biotic and abiotic stress responses [18]. The *MPK3/6* induced ethylene synthesis through the ethylene signaling pathway to activate *ERF1A*, *ERF1*, and *ORA59* transcriptionally, subsequently influencing the defense of genes in *Arabidopsis* [19]. Meanwhile, our study found that scale insect stimulation could activate both ET and JA pathways, and the response genes of ET and JA also indicated that there might be crosstalk between them. The JA signaling pathway was central defense signal during resistance to insect attack, inducing massive defense-related genes and biosynthesis of terpenes, flavonoids [20]. In our study, the JA signaling pathway was activated in *C.sasanque*, and we identified six significantly up-regulated genes after infestation by scale insects. JAZ interacting *MYC2* transcription factors to activate JA responses participate in plant resistance against insects. MYCs can impair the function of insect digestive system and control the biosynthesis of defense secondary metabolites [21]. For example, *Arabidopsis* resists insects by inducing toxic metabolites glucosinolates, and *MYC2/MYC3/MYC4* was required for direct transcription of glucosinolates biosynthesis [22]. Other hormones also was regulated significantly in response to scale insects, such as ABA, SA, CK. Thereby illustrating growth and defense hormones interconnectedness in plant resistance to scale insects networks. Furthermore, the showed that this crosstalk between hormones provided great regulatory potential for activating multiple defense mechanisms in varying combinations.

### Transcription factor played a role in the resistance of scale insects

Our results showed that scale insects induced the 17 AP2/ERF transcription factors in *C. sasanqua* (Fig. 7)*.* The transcription factor of this family induced secondary metabolites by activating genes to enhance biotic stress response and tolerance in plants or act as response factors to regulate various growth processes of plants [23]. *ERF5* through differentially regulated chitin and other defense pathways in plant response [24]. And transgenic *Arabidopsis* expressing *SaERF1* also enhanced defense gene transcript levels [25]. These 17 ERF family genes might strengthen the plant to resist scale insects by regulating hormones or increase the plants ability to fight abiotic stress. Previous studies showed that the MYB family transcription factors are essential in defense responses against insects [26]. *GsMYB15* enhanced resistance to *Helicoverpa armigera* in transgenic *Arabidopsis* [27]*. OsMYB30* regulated the expression of *OsPALs* to confer brown planthopper resistance in rice [12]. Moreover, *AtMYB44* held the expression of EIN2 to repress the reproduction of the green peach aphid (*Myzus persicae* Sulzer) and diamondback moth (*Plutella xylostella* L.) [28]. We also found *MYB44* in *C. sasanqua* in transcriptome data and deemed that the gene might have the same function*.*

The family of WRKY transcription and NAC transcription factors were prominent expressions. WRKY transcription factors were known to have a significant role in plant defense against sap-sucking insects. Tobacco *WRKY3* and *WRKY6* could coordinate responses to hornworm infestation and create induced resistance through regulation of the biosynthesis of phytohormones [29]. *AtWRKY33* negatively modulated plant defense against whitefly in *Arabidopsis* [30]. The part of the WRKY family genes was related to plant defense hormones, such as JA, SA, and other signaling pathways. Our data also suggested that the WRKYs positively modulate plant defense against scale insects through interaction with the MAPK signal transcription pathways in *C. sasanqua*. NAC transcription factors were reported to be involved in the responses of plants to various biotic stresses [31]. For example, bean pyralid larvae induced NAC genes in soybeans [32]. We assumed that WRKY and NAC family-related genes might cause response insect resistance in *C. sasanqua*.

### Secondary metabolism played a key role in the resistance of scale insects

Plant defenses rely on the flexible expression of genes in the secondary metabolism. The phenylpropanoid and flavonoid pathways were crucial defense-responsive pathways in insect affected tea plants [2]. Scale insect attacks caused the gene of phenylalanine pathway up-regulation in *C. sasanqua*. The lignin was mainly produced from the metabolism of the phenylalanine pathway. Previous research reports the involvement of lignin in insect resistance. The accumulation of lignin improved the thickness of cell walls, making it difficult for insects to feed on plants. It also helped transport water, minerals, and organic over long distances, strengthening the ability of plants to resist insects. In soybeans, lignin accumulation improved resistance to aphids [33]. *PAL* affected lignin synthesis in the phenylalanine pathway. The lignin biosynthesis was activated by *RcPAL* in castor and improved the resistance of castor beans [34]. In the case of tea mosquito bug (piercing-sucking pest) infestation of a tea plant, the level of *PAL* increases. We found significant *PAL* significant up-regulation in the transcriptome data. In addition, we also found the accumulation of scopolin. And lignin and scopolin might be essential in the plant resistance response to insects.

Flavonoid metabolism was mentioned as a defensive pathway preventing plants from insect invasion. The flavonoid metabolites could directly inhibit insect feeding, development, and egg-laying, overexpression concentrations of flavonoids are thought to be toxic to insects [35, 36]. Our results showed that *C. sasanqua* was infested to produce secondary metabolism by the scale insects, including afzelechin, gallocatechin, quercetin, kaemferol, myricetin, and catechin. Afzelechin, gallocatechin, and quercetin formed proanthocyanidins by converting *LAR* [37]. In *Arabidopsis*, kaempferol-3,7-dirhamnoside was indentified as a defensive metabolite, after kaempferol-3,7-dirhamnoside significant reduction, increased *MYB75* susceptibility regulated anthocyanin and flavonol levels which enhances plant resistance to *Pieris brassicae* [38]. In our study, the gene expression of the phenylalanine pathway and flavonoid metabolites revealed a striking up-regulation. Secondary metabolism genes should play an important role in the downstream network response of *C. sasanqua*.

### Energy-related metabolism may played a role in the defense of scale insects

The scale insects infected plants and induced the expression of genes associated with carotenoid biosynthesis and galactose metabolism. Photosynthesis was involved in plant defense responses and physiological function as a remedy for carbon loss. Carotenoid biosynthesis was closely bound up with light capture, photoprotection, and stabilization of the photosynthetic apparatus and involved in many stress responses [39–41]. *Nephrolepis biserrata* was infested by the scale insect *Coccus hesperidum* significantly decreased carotenoid accumulation [42]. In poplars, the carotenoid biosynthesis genes were enriched, and down-regulation reduced the availability of essential nutrients and thus compromised herbivore performance [43]. Our transcriptomic data showed that *NCED*, *CYP707A*, and *BG1* showed up-regulation or down-regulation significantly and they (Fig. 6). *NCED* was identified to modulate plant development and participate in plant response to drought stress [44, 45]. *BG1* was a key enzyme involved in synthesizing scopolamine in the phenylpropane pathway, validating our previous conclusion that the *C. sasanqua* defense systems might exist as a network involving interactions. We speculated that the photosynthesis rate was influenced during scale insects attack plants due to activated carotenoid biosynthesis, contributing to the adaptive response mechanisms toward scale insect resistance.

Plant defense was costly and sacrificed growth and energy to support and aid complex defense responses. The *Aphis gossypii* Glover and *Acyrthosiphon gossypii* Mordvilko enhanced the differential expression of galactose metabolism in cotton. Metabolites from galactose metabolism strongly influence plant defense function [46]. In resistant rice cultivars, the responses of brown planthoppers, galactose metabolism as primary metabolism, and energy metabolism were significantly affected [47]. We found that *GOLS* and *GSG* produced remarkable changes in galactose metabolism. Most of the changed genes were involved in the resistance stress of plants. We assumed that galactose metabolism might participate in the resilience of *C. sasanqua* and energy redistribution in response to scale insects.

## Materials and methods

### Observe the occurrence rule of scale insects and plant material and collection

From March 2021 to March 2022, we observed scale insects on the *C. sasanqua* of Southwest Forestry University, Kunming, Yunnan Province, China (102°10′ ~ 103°40′E, 24°23′ ~ 26°33′N) with an altitude of about 1990 m and a subtropical plateau monsoon climate. The average annual temperature is 15 ℃, and the annual precipitation is 1035 mm in Kunming. The eight *C. sasanqua* infected by scale insects were selected, and the four orientations, north, south, east, and west, were recorded and distinguished. The life patterns of scale insects were observed at ten-day intervals, the branches of different heights and directions of growing scale insects were clipped, and the growth process of insects was monitored and recorded.

The *Camellia sasanqua* Thunb. was collected in Kunming, Yunnan Province, China, and identified by Professor LongQing Chen, a botanist. A voucher specimen (No. 20211216) was deposited at the herbarium of the School of Landscape Architecture and Horticulture Sciences, Southwest Forestry University, China. The well-grown leaves without signs of insect or disease infestation were used as a control. Each sample included three replicates. The samples were immediately frozen in liquid nitrogen and stored at -80 ℃ for transcriptome sequencing analysis.

### Making specimen of scale insect and observation of microscopic

After removing the white covering, a complete scale insect was selected as the specimen with a picking needle. The chosen scale insects were placed in a 2ml centrifuge tube containing 5% NaOH and soaked overnight until the insects bodies were transparent. Transfer the scale to distilled water with a brush and wash 2–3 times. After cleaning, the dehydration was carried out with 30%, 50%, 70%, 90%, 95%, and anhydrous ethanol. The scale insects were placed in the middle of the slide, the resin was added, the decline was covered and gently pressed, and then dried at 35–40 ℃ after natural drying. The specimen was stored after the slide specimen was made, and the slide specimen was labeled with information such as the type of insect and its organs, production location, producer, and production time.

### Validation of DEGs by qRT-PCR

The 17 genes were randomly selected from the transcriptome data based on their differential expression. The *Actin* of *camellia sinensis* was used as an internal reference and amplified with the target gene, and the primers (Table S2) were designed using NCBI primer-BLAST (https://www.ncbi.nlm. nih.gov/tools/primer-blast/). The qPCR was performed on the LightCycler 480 II (Roche, Shanghai, China). The 10 µL ChamQTMSYBR qPCR Master Mix, 0.6 µL upstream primer, 0.6 µL downstream primer, 2 µL cDNA, and 6.8 µL ddH_2_O were used as the qRT-PCR reaction. The qRT-PCR reactions were performed in 96-well plates using the ABI7500 fast Real-Time PCR system (Applied Biosystems, Foster City, CA, USA) with the SYBR Premix ExTaq™ Kit (Takara, Dalian, China). Three replicate reactions of each sample were assayed. Furthermore, the expression level analyses of these genes were calculated using the 2^−∆∆CT^ method.

### RNA extraction, library construction, and sequencing

The total RNA of six samples (three biological replicates per treatment group) was isolated using the CTAB method [48]. We analyzed the degradation and contamination of RNA extracts with 1% agar gel electrophoresis. The OD260/280 was about 2.0, and the OD260/230 was between 1.9 and 2.2. DNA fragments from samples were synthesized using the Tiangen Quant cDNA reverse transcription kit (Tiangen, Beijing, China). Then, the qualified RNAs were used for transcriptome sequencing using the Illumina Novaseq 6000 platform.

### Differential expressed genes analysis

After obtaining high-quality clean data, assemble all clean data from scratch using the Trinity (https://github.com/trinityrnaseq/trinityrnaseq/wiki). All of the genes were compared with public databases, including the NR database (http://www.ncbi.nlm.nih.gov/), Swiss-Prot database (http://www.expasy.ch/sprot), the COG (http://www.ncbi.nlm.nih.gov/COG/), Pfam (http://pfam.xfam.org/), GO database (http://www.geneontology.org) and KEGG database (http://www.genome.jp/kegg). DEGs analysis of plant samples was performed using the DEGseq2 package. *p-*value < 0.05 and | log_2_(fold change) |≥ 1 was set as the threshold for significantly differential expression. Two gene sets were defined as DEGs of normal leaves and the leaves infested.

### GO function annotation and KEGG enrichment analysis of DEGs

GO annotation analysis was performed on all identified DEGs to classify further and explore differential gene trends. GO terms were usually classified into three categories, biological process (BP), cellular component (CC), and molecular function (MF). Meanwhile, All DEGs were mapped to the KEGG database, with a threshold value (*p*-value) < 0.05 set to evaluate significantly enriched GO Annction and the KEGG pathways, and used the Hochberg (BH) multiple test correction to analyze KEGG enrichment genes.

### Identification of transcription factor analysis

The transcription factor database AnimalTFDB and PlantTFDB was used to predict transcription factors. According to the prediction results, the transcription factor families were counted, and related to insect resistant transcription factors were screened.

#### Measurement of anthocyanin content

After the leaves were ground into powder with liquid nitrogen, about 2 g of the powder was weighed into 20 mL, 0.1% methanol, and 1 ml of ice acetic acid were added, and the extract was leachated under dark light for eight hours in indoor temperature. After a 0.22 μm filter head filtered the extract, the absorbance at 530 nm, 620 nm, and 650 nm were determined by ultraviolet spectrophotometer.

The formula is as follows: OD = (OD_530_-OD_620_)-0.1(OD_650_-OD_620_).

## Conclusions

**Fig. 9:**
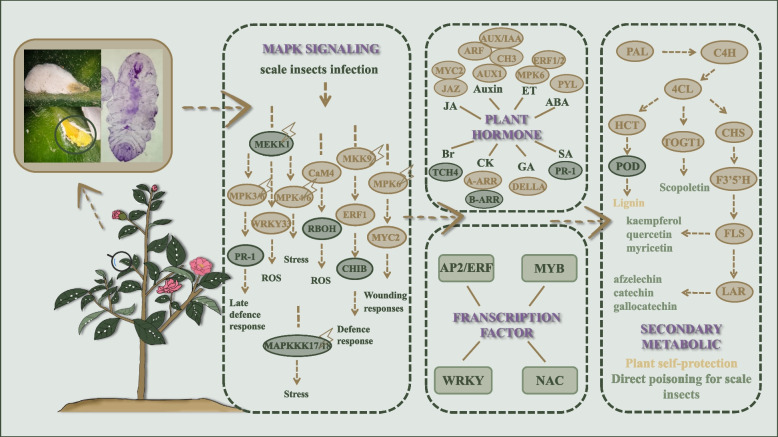
Response mechanism of *C. sasanqua* in response to insect stimulation

The transcriptomic analysis revealed the molecular mechanism of *C. sasanqua* in response to scale insects. Our data found that scale insect infestation activated the MAPK signaling pathway, plant hormones, transcription factors, and genes related to secondary metabolism. Therefore, we deemed that the response of *C. sasanqua* to scale insects is a complex defense network system (Fig. 9). Our findings provided new evidence of the relationship between *C. sasanqua* and scale insects and helped improve our understanding of the roles of plant hormones, transcription factors, and secondary metabolism in mediating plant defense against scale insect infestation.

### Supplementary Information

**Additional file 1:** **Fig S1. **Correlation analysis. N: normal leaves, SL: the leaves infected by scale insects. **Fig S2. **qRT-PCR validation of DEGs. **Table S1. **The sequenced result in normal leaves and leaves infected by scale insects. **Table S2** Primer sequence of qPCR.

## Data Availability

No datasets were generated or analysed during the current study.

## Abbreviations

KEGG: Kyoto Encyclopedia of Genes and Genomes
DEGs: Differentially expressed genes
PCA: Principle component analysis
GO: Gene Ontology
MAPK: Mitogen-activated protein kinase
TF: Transcription factors
