# Supplementary material for: Transcriptome analysis reveals the potential mechanism of the response to scale insects in Camellia sasanqua Thunb
Source: BMC Genomics. 2024 Jan 24;25:106. doi: 10.1186/s12864-024-09980-y (PMC10807073; doi:10.1186/s12864-024-09980-y)
Supplement: Supplementary file 1 — Additional file 1: Fig S1. Correlation analysis. N: normal leaves, SL: the leaves infected by scale insects. Fig S2. qRT-PCR validation of DEGs. Table S1. The sequenced result in normal leaves and leaves infected by scale insects. Table S2 Primer sequence of qPCR. [file 12864_2024_9980_MOESM1_ESM.docx]

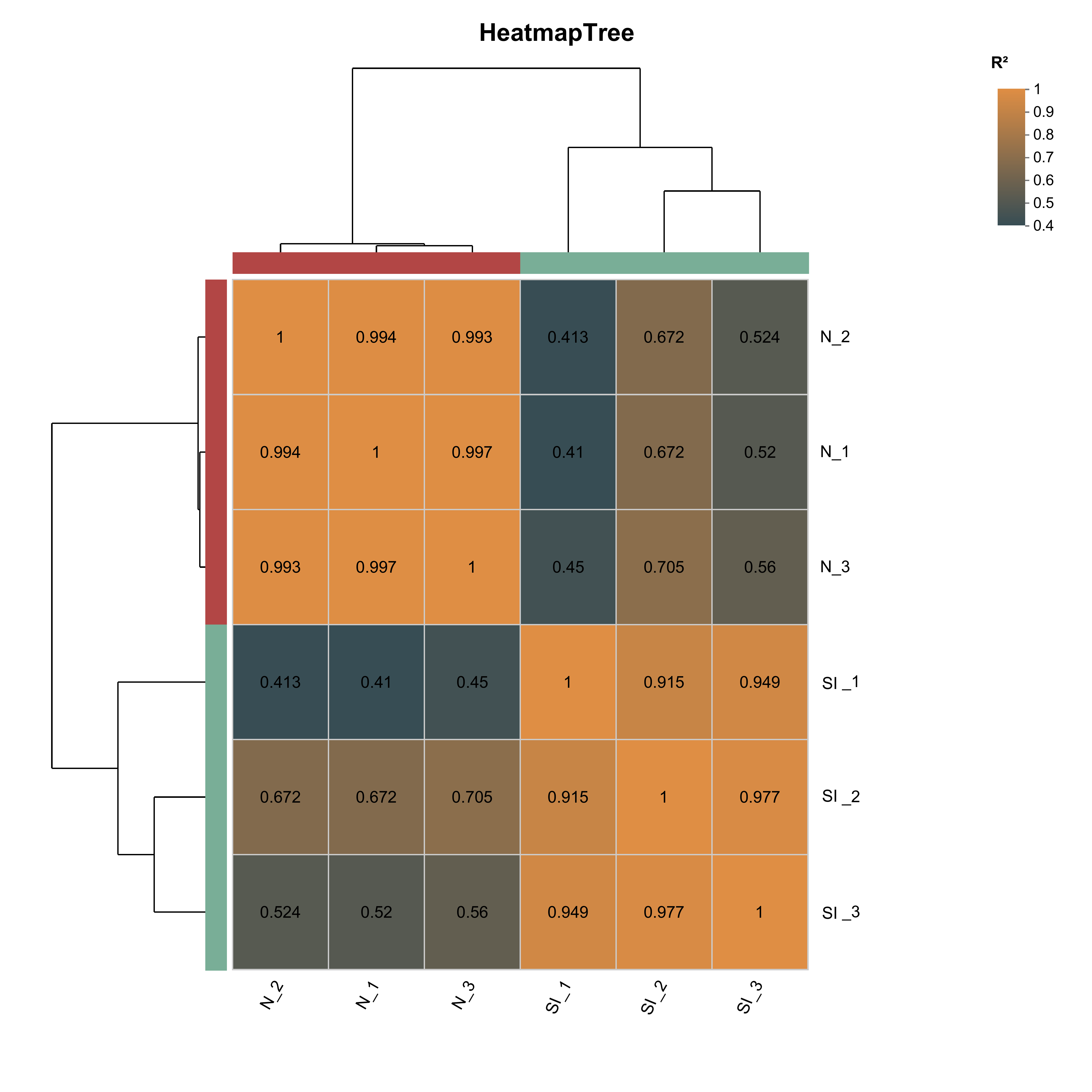


**Fig S1.** Correlation analysis. N: normal leaves, SL: the leaves infected by scale insects.


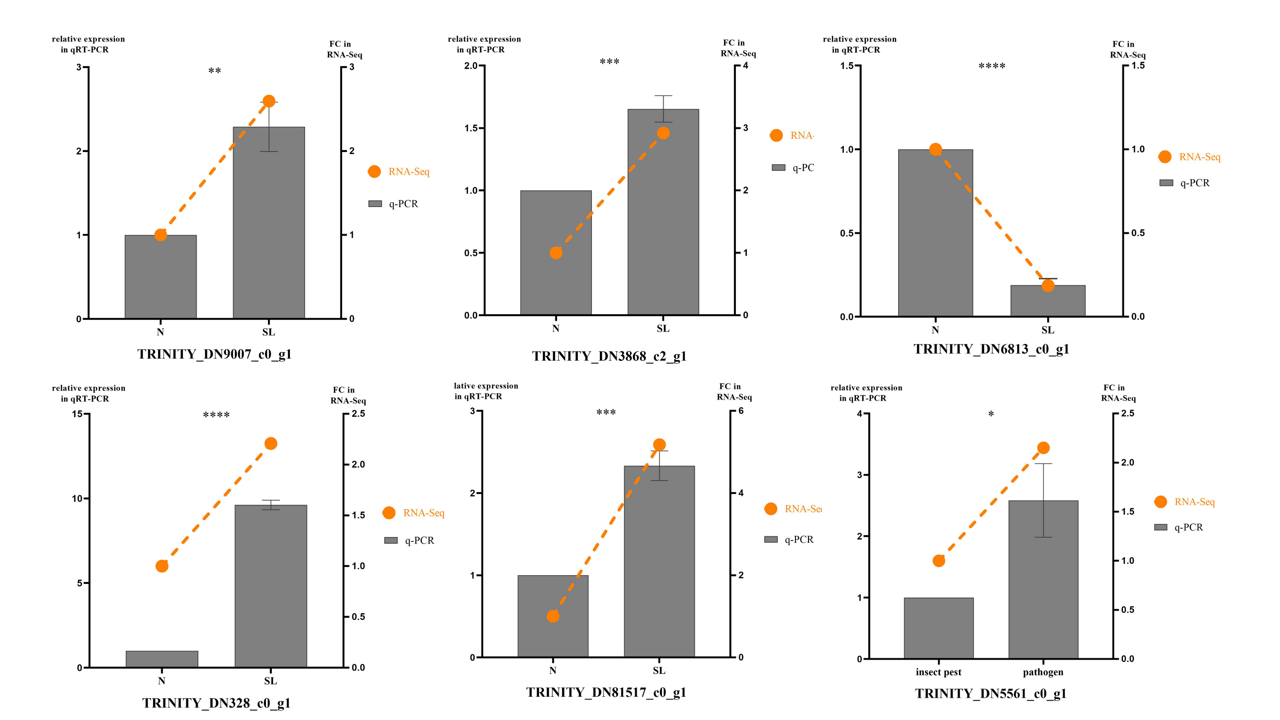

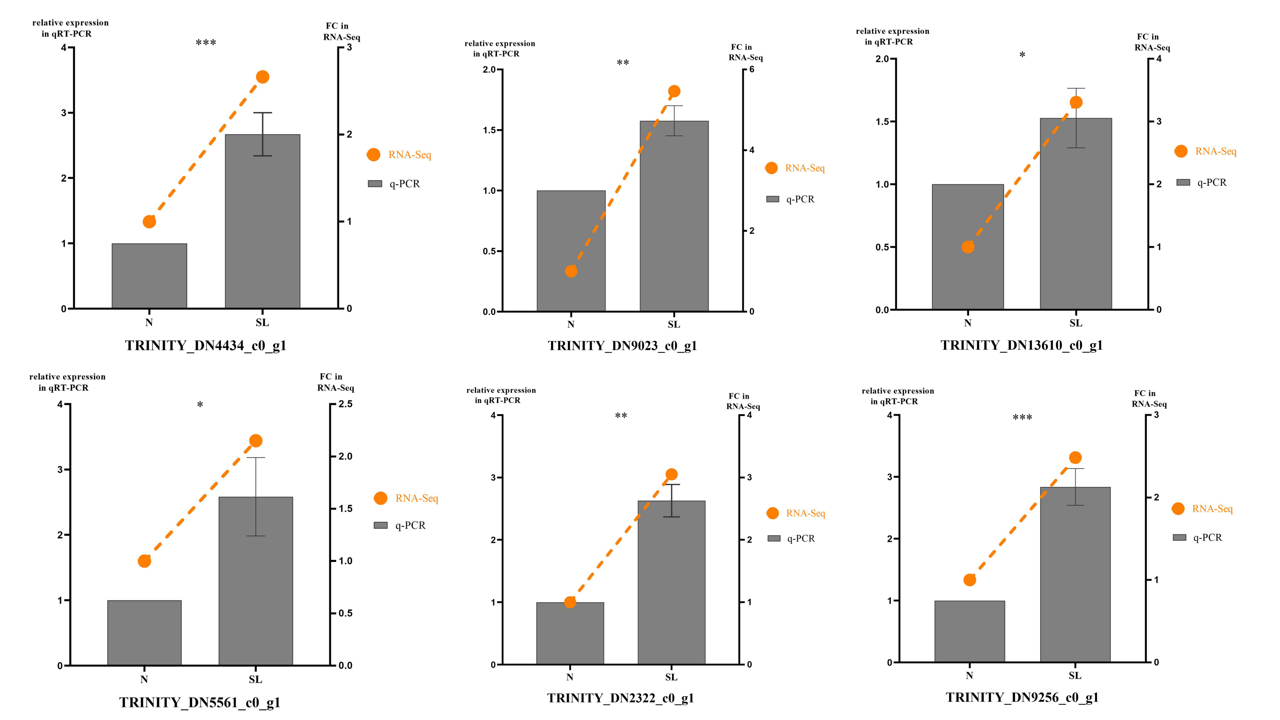

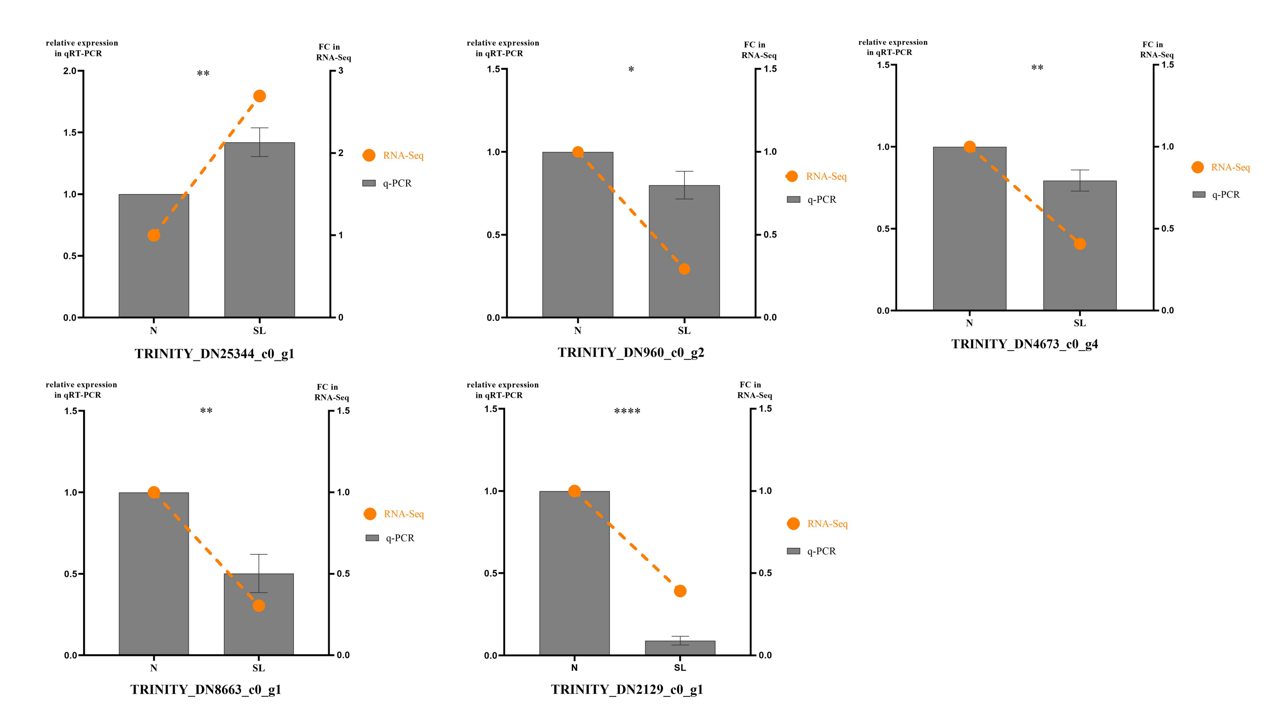


**Fig S2.** qRT-PCR validation of DEGs.

**Table S1** The sequenced result in normal leaves and leaves infected by scale insects

| Sample | Raw reads | Raw bases | Clean reads | Clean bases | Error rate(%) | Q20(%) | Q30(%) | GC content(%) |
| --- | --- | --- | --- | --- | --- | --- | --- | --- |
| N_3 | 47934388 | 7190158200 | 46731398 | 6814824801 | 0.0256 | 97.7 | 93.65 | 45.26 |
| N_2 | 44976980 | 6755488868 | 44534116 | 6568728180 | 0.0255 | 97.81 | 93.75 | 45.39 |
| N_1 | 47049114 | 7057367100 | 45266876 | 6628607147 | 0.0264 | 97.37 | 92.91 | 45.38 |
| SI_3 | 47004948 | 7050742200 | 45679808 | 6670941894 | 0.0259 | 97.61 | 93.45 | 44.83 |
| SI_2 | 44211966 | 6631794900 | 42458936 | 6213202303 | 0.0261 | 97.5 | 93.29 | 45.24 |
| SI_1 | 42731784 | 6409767600 | 41533976 | 6046633366 | 0.0261 | 97.51 | 93.21 | 45.17 |

**Table S2** Primer sequence of qPCR

| Primer name | Forward sequence (5’-3’) | Backward sequence (5’-3’) |
| --- | --- | --- |
| ACTIN | GCCATCTTTGATTGGAATGG | GGTGCCACAACCTTGATCTT |
| TRINITY_DN8186_c0_g1 | CGACATTGTTGAGAGCTTTG | CCTCTTCAACCACAGATACC |
| TRINITY_DN9023_c0_g1 | GCTTTTGTTGATGCTGACAA | TTCCTCCACTGTCTCTTGTA |
| TRINITY_DN81517_c0_g1 | AGAAAATATGGTCTCGGAGC | GACAGGAACCAACCTTACTT |
| TRINITY_DN9007_c0_g1 | AAGGAATGTCCAATCACTCC | GGCAAATCAAAGTTGATCCC |
| TRINITY_DN328_c0_g1 | TTGCCCTGTTACTGATAACC | TGCAGAACTCAAGATCACTC |
| TRINITY_DN9256_c0_g1 | TTCGACAACGCTTACTACAA | GCTAAGGTTTCCCATCTTCA |
| TRINITY_DN25344_c0_g1 | CACTCCACTCCAATTTCTCA | CAAAAGCGGTGTTGATGTAA |
| TRINITY_DN5561_c0_g1 | GGCCTCACTATGTTAAGGTT | GTTGTTGAATGCTACTGCTC |
| TRINITY_DN3868_c2_g1 | TTGAAGCCCTTCTACTCAAC | AAAACCACCTGTTTGGAGAT |
| TRINITY_DN13610_c0_g1 | ATGGAAAGTATCATGGGTCG | CTTACGTACTTCCTCATCGG |
| TRINITY_DN4434_c0_g1 | TTTTAGTTGGAAGCGCCTAT | CGTTGACCGAGTTTATCTCT |
| TRINITY_DN2322_c0_g1 | ACAAGCTCCCAATTGTTGTA | ACATTTTGGACCCATACCTC |
| TRINITY_DN6813_c0_g1 | ATGGATTCATTGATCTCCGC | TCAAACAATCTCTCCACGAG |
| TRINITY_DN960_c0_g2 | ATGAAAACCCAGATCAGTCC | CTTTTGGCATCAGGTTCATC |
| TRINITY_DN2129_c0_g1 | TGGAAAACGAACCGATTTTG | TTTTGACCTCTCGCTTAACA |
| TRINITY_DN8663_c0_g1 | CCACTTTACATGTGCATCAG | ACCAGTCTGGCGACATAATA |
| TRINITY_DN4673_c0_g4 | TGCATACACTCACTTCAACA | ACAGTTCACCATCGAAATCA |
